# Rethinking mitochondrial isolation for the next generation of mitochondrial therapies

**DOI:** 10.3389/fphys.2026.1975105

**Published:** 2026-10-06

**Authors:** Ethan A. Simpson, Celina Phan, Jason P. Acker

**Affiliations:** 1 Department of Laboratory Medicine and Pathology, University of Alberta, Edmonton, AB, Canada; 2 Department of Biomedical Engineering, University of Alberta, Edmonton, AB, Canada; 3 Canadian Blood Services Innovation, Canadian Blood Services, Edmonton, AB, Canada

**Keywords:** bioprocessing, isolation, mitochondria, oxidative injury, stabilization

## Abstract

Mitochondrial isolation is fundamental to the study of mitochondrial biology and is increasingly relevant to emerging therapeutic applications such as mitochondrial transplantation. However, isolation procedures can substantially influence mitochondrial yield, purity, structural integrity, and bioenergetic function, making method selection an important determinant of experimental and translational outcomes. This review examines current mitochondrial isolation strategies, including physical disruption, chemical permeabilization, emerging microfluidic and automated dissociation technologies, and downstream separation approaches such as differential centrifugation, density-gradient centrifugation, filtration, and immunoaffinity enrichment. Each approach offers distinct advantages and limitations depending on the biological source, processing requirements, and intended application. Conventional methods remain widely used because of their accessibility and flexibility, but may introduce operator variability, mechanical stress, contamination, or loss of mitochondrial function. More selective purification strategies can improve sample purity, although often at the cost of additional processing time, technical complexity, and reduced recovery. Emerging technologies, including standardized mechanical dissociation and microfluidic disruption, may provide greater control over mitochondrial release, but require broader validation across tissues and sample volumes. A persistent challenge across isolation workflows is balancing mitochondrial recovery with preservation of organelle integrity and function. Future progress will require standardized, scalable isolation strategies that consistently preserve mitochondrial quality across different biological sources and downstream applications. Continued refinement of these approaches will be essential for advancing mitochondrial research and supporting the development of mitochondrial-based therapies.

## 1 Introduction

Early observations of structures now recognized as mitochondria date to the nineteenth century. Theodor Schwann in 1839 and Friedrich Gustav Jakob Henle in 1841 described granular structures within striated muscle that were later interpreted as mitochondrial structures (Schwann, 1839; Henle, 1841; Glancy and Balaban, 2021; Glancy et al., 2021). Albert von Kölliker subsequently provided a more detailed description of these structures in striated muscle in 1857 and later examined their distribution across muscle types and their behavior under different osmotic conditions, including swelling (Kölliker, 1857; Glancy and Balaban, 2021; Glancy et al., 2021). Several decades later Richard Altmann described these structures in greater detail and termed them “bioblasts” in 1890 and later termed “mitochondria” in 1898 by Carl Benda (Ernster and Schatz, 1981; Glancy et al., 2021). Mitochondria have since been recognized as central regulators of cellular energy metabolism, leading to their well-known designation as the “powerhouses of the cell” (Ernster and Schatz, 1981; Nelson and Cox, 2021). Since then, the understanding of mitochondrial function has expanded considerably, revealing roles that extend far beyond adenosine triphosphate (ATP) production. For instance, mitochondria also contribute to calcium homeostasis, redox signaling, apoptosis, intermediary metabolism, innate immune responses, steroid synthesis, and cellular stress adaptation (Hajnóczky et al., 2006; Li et al., 1997; Ishikawa et al., 2009; Stocco, 2001). Their semi-autonomous nature, dynamic morphology, and dependence on both mitochondrial and nuclear genomes position mitochondria as highly integrated organelles that are essential for maintaining cellular and tissue homeostasis (Chan, 2006; Wallace, 2005; Alberts et al., 2022).

Mitochondrial dysfunction arises when mitochondria are unable to adequately maintain cellular bioenergetic, metabolic, or signaling functions (Wallace, 2005). This dysfunction may result from pathogenic variants in mitochondrial (mtDNA) or nuclear DNA, impaired electron transport chain activity, altered mitochondrial dynamics, defective mitophagy, excessive reactive oxygen species production, disrupted calcium homeostasis, or environmental and metabolic stressors (Wallace, 2005; Chan, 2006; Brand, 2010; Youle and Narendra, 2011). As cellular energy demands vary across tissues, mitochondrial dysfunction often produces heterogeneous clinical manifestations, particularly in organs with high bioenergetic requirements, including the brain, skeletal muscle, heart, liver, kidney, and endocrine system (Wallace, 2005; DiMauro and Schon, 2003). Consequently, mitochondrial dysfunction has been implicated not only in primary mitochondrial diseases, including Leigh syndrome, mitochondrial encephalomyopathy with lactic acidosis and stroke-like episodes (MELAS), myoclonic epilepsy with ragged-red fibers (MERRF), and Leber hereditary optic neuropathy (LHON), but also in broader pathological contexts including neurodegenerative disorders, cardiovascular disease, metabolic syndrome, cancer, aging-related decline, and inflammatory disease (DiMauro and Schon, 2003; Wallace, 2005). Although individually rare, primary mitochondrial diseases collectively represent one of the most common inherited metabolic disorders, with previous studies estimating the global prevalence to be 1 in 5000 births (Chinnery et al., 2000; Wen et al., 2025). More recently, a 2022 study from Ontario reported a prevalence of approximately 2.5 cases per 10,000 individuals (~1 in 4,000) (Buajitti et al., 2022). Additionally, it is estimated that 1 in 200 healthy individuals carries a pathogenic mtDNA mutation (Elliott et al., 2008; Wen et al., 2025). These disorders are characterized by considerable clinical heterogeneity resulting from the diverse genetic and functional defects that impair mitochondrial homeostasis (DiMauro and Schon, 2003; Wallace, 2005).

Despite major advances in mitochondrial biology, therapeutic development has lagged behind mechanistic discovery (Gorman et al., 2016; Chinnery, 2023). Current management of many mitochondrial disorders remains largely supportive and symptom-directed, often involving nutritional optimization, exercise therapy, vitamin or cofactor supplementation, antioxidant administration, and treatment of organ-specific complications (Gorman et al., 2016; Ng and Turnbull, 2016). While these approaches may improve quality of life or stabilize selected clinical manifestations, they rarely address the underlying mitochondrial defect (Gorman et al., 2016; Chinnery, 2023). Recent progress, including the approval of omaveloxolone (sold as Skyclarys^®^) for Friedreich’s ataxia, demonstrates that disease-modifying therapies targeting mitochondrial dysfunction are achievable (Lynch et al., 2021; Lee, 2023; Chinnery, 2023). However, broadly effective, mechanism-based therapies remain limited, and many investigational approaches continue to face significant challenges related to tissue delivery, genetic heterogeneity, disease timing, biomarker validation, and clinical trial design (Chinnery, 2023).

Emerging therapeutic strategies increasingly aim to target mitochondrial dysfunction directly rather than simply managing downstream clinical manifestations. These include mitochondrial-targeted antioxidants, cardiolipin-stabilizing peptides, NAD+ augmentation, mitophagy modulators, metabolic rewiring strategies, gene- and RNA-based therapies, mitochondrial transfer approaches, and organelle-targeted drug delivery platforms (Chinnery, 2023; Ng and Turnbull, 2016; Wen et al., 2025). Of particular interest, mitochondrial transplantation has emerged as a promising therapeutic strategy to restore cellular bioenergetics through the delivery of functional mitochondria to damaged or metabolically compromised cells (Luo et al., 2024; Kubat et al., 2025; Miao et al., 2025). Preclinical studies have demonstrated that mitochondrial transplantation can improve mitochondrial function, tissue repair, and cell survival across a range of disease models (McCully et al., 2009; Cowan et al., 2016; Hayakawa et al., 2016).

Despite encouraging preclinical results, significant challenges remain before widespread clinical application of mitochondrial transplantation can be achieved. Regardless of the intended downstream application, the successful use of isolated mitochondria depends on obtaining organelles that retain their structural integrity, bioenergetic function, and physiological competence following extraction from their cellular environment (Picard et al., 2011; Frezza et al., 2007). Achieving this remains challenging, as currently available isolation methods often require trade-offs between mitochondrial yield, purity, structural integrity, and functional performance. Furthermore, the absence of standardized isolation protocols and the limited compatibility of many existing methods with long-term preservation and large-scale production continue to hinder both experimental reproducibility and clinical translation (Picard et al., 2011; Frezza et al., 2007; Liao et al., 2020).

This review provides a comprehensive overview of current mitochondrial isolation methodologies, evaluating their impact on mitochondrial quality, function, and downstream application. Furthermore, we highlight key methodological limitations, identify opportunities for optimization and standardization, and discuss future directions for developing robust, scalable isolation strategies capable of supporting both experimental and clinical applications.

## 2 Factors influencing isolation strategy

The choice of mitochondrial isolation strategy will often depend on the source material, as tissue type and cell type can influence mitochondrial abundance, accessibility, tissue fragility, and susceptibility to processing-related damage. Highly accessible sources such as blood are attractive for their minimally invasive sampling, but their mitochondrial content differs across cell types. While abundant, mature erythrocytes are a poor mitochondrial source because they lack mitochondria, nuclei, and other organelles (Bratosin et al., 2001; Moura et al., 2025). In contrast, platelets are readily available from whole blood and contain functional mitochondria, although their mitochondrial content is relatively low, with most platelets containing approximately four to ten mitochondria (Melchinger et al., 2019; Hayashi et al., 2011; Moura et al., 2025). Despite the low mitochondrial content, platelets present as an attractive source because they are routinely prepared as transfusable blood components from whole-blood donation or apheresis, but their short shelf life creates persistent inventory-management challenges (Haran et al., 2024). This low per-platelet mitochondrial content is offset by the large number of platelets present in a transfusable platelet component. Platelet products can range from 2.0 to 4.0 × 10^11^ platelets, which would correspond to approximately 8.0×10^11^ to 4.0×10^12^ platelet-derived mitochondria, assuming four to ten mitochondria per platelet (Ohto et al., 2025). In a 2024 retrospective study of blood-component wastage, platelets represented 91.6% of discarded components over a five-year period, and expired shelf life was the most common reason for discard (Haran et al., 2024). This suggests that platelet-derived mitochondria may potentially be an underutilized whole blood-derived mitochondrial source. Lastly, leukocytes provide another accessible blood-derived source, but they are heterogeneous, and mitochondrial measurements can vary according to immune-cell subtype (Moura et al., 2025).

Beyond blood, solid tissues may also be used as a source of mitochondria. Heart, liver, and skeletal muscle are metabolically active tissues with high mitochondrial abundance (Moura et al., 2025). Adult cardiomyocytes, for example, have very high energetic demand, and have approximately 3000–8000 mitochondria per cell. Similarly, hepatocytes also contain abundant mitochondria, with three-dimensional reconstruction studies estimating approximately 1,000 to 1,700 mitochondria per hepatocyte (Moura et al., 2025). Lastly, skeletal muscle cells contain 120 to 160 mitochondria (Moura et al., 2025). Despite the mitochondrial abundance, these solid tissues introduce different practical constraints. Unlike whole blood, they are generally less accessible, often require biopsy or surgical material, and may be more vulnerable to processing-related variation (Acín-Pérez et al., 2023). Therefore, source selection should balance mitochondrial abundance against accessibility, tissue fragility, processing feasibility, and the intended downstream application.

## 3 Cell disruption methods

A critical step in mitochondrial isolation is the controlled disruption of cells or tissues to release mitochondria while preserving their structural and functional integrity. Cell disruption methods differ in the mechanisms by which they disrupt the plasma membrane, ranging from physical approaches that apply mechanical forces to chemical strategies that alter membrane stability through detergents or osmotic conditions. Consequently, the choice of disruption method has a direct influence on mitochondrial yield, purity, structural integrity, and bioenergetic function, making it a key determinant of overall isolation quality. For a broader comparison of the strengths, limitations, and practical applications of each mitochondrial isolation technique, refer to **Table 1**.

**Table 1:** Cell disruption methods for mitochondrial isolation: summary, applications, and limitations.

Disruption approach	Processing efficiency*	Reproducibility	Best-fit use	Main limitation
Dounce homogenization (Acín-Pérez et al., 2023; Clayton and Shadel, 2014; Dounce et al., 1955)	Moderate	Moderate, but operator-dependent	Cultured mammalian cells and relatively soft tissues	Manual stroke number, pestle clearance, and tissue type strongly affect disruption efficiency
Potter-Elvehjem homogenization (Frezza et al., 2007)	Moderate	Moderate to high when motor-driven	Solid tissues such as liver, skeletal muscle, heart, and kidney	Requires optimization of pass number, rotation speed, tissue-to-buffer ratio, and temperature
Rotor-Stator homogenization (Idell-Wenger et al., 1982; Goldberg, 2008; Preble et al., 2014)	High	Moderate	Fibrous or mechanically resistant tissue samples requiring rapid breakdown	Strong shear can cause over-disruption, heating, and mitochondrial damage if not tightly controlled
French press/pressure cell (French and Milner, 1955; Watson et al., 1970; Fukami and Salganicoff, 1973)	Moderate	Moderate to high	Yeast, microorganisms, algae, platelets, or resistant source materials	Pressure must be optimized. Excess pressure risks organelle damage, while insufficient pressure leaves incomplete disruption
Nitrogen cavitation (Kristián et al., 2006; Younis et al., 2023)	Moderate	High	Sensitive cells or tissues where preservation of mitochondrial respiration is a priority	Requires pressure equipment and optimization of pressure, equilibration, and decompression conditions
Bead beating/bead milling (Mogren et al., 1974; Diekert et al., 2001; Mendoza-Martínez et al., 2026)	High	Low to moderate	Yeast, fungi, bacteria, algae, and rigid cell-wall samples	Harsh impact forces and heat generation can compromise mitochondrial integrity
Sonication (Wood and Loomis, 1927; Rahman et al., 2018; Shehadul Islam et al., 2017)	High	Low to moderate	Resistant samples or mitochondrial protein extraction workflows	Cavitation, heat, and membrane fragmentation can damage mitochondria
Electroporation (Tsong, 1991; Kotnik et al., 2019; Bahnemann et al., 2014)	Moderate	Moderate to high	Cultured cells where electrical permeabilization can be controlled; currently best treated as an experimental disruption approach rather than a routine mitochondrial isolation method	Requires careful optimization of electric-field strength, pulse duration, pulse number/frequency, waveform, electrode configuration, and sample properties; excessive exposure may compromise mitochondrial integrity or function
Enzymatic pretreatment/assisted disruption (Gross et al., 2011; Garcia-Cazarin et al., 2011; Gregg et al., 2009; Koncsos et al., 2018; Lai et al., 2019; MacDonald et al., 2019)	Moderate	Moderate	Serine proteases such as subtilisins and trypsin for muscle preparations; collagenases and dispase for tissue dissociation; cell-wall-degrading preparations such as Zymolyase for yeast	Generally combined with mechanical disruption; protease exposure can alter mitochondrial protein detection, and the sequence of digestion and disruption varies between protocols
Saponin-assisted mitochondrial release (Salabei et al., 2014; Niklas et al., 2011; Okutani et al., 2025)	Moderate	High if carefully titrated	Selective plasma membrane permeabilization in cells or permeabilized fibers	Highly concentration-, time-, temperature-, and sample-dependent
Cholesterol-dependent cytolysin pretreatment (Shibata et al., 2015; Salabei et al., 2014; Okutani et al., 2025; Endo et al., 2026)	Moderate	Moderate	Streptolysin O-assisted mitochondrial isolation from cultured mammalian cells; perfringolysin O for respiration measurements in permeabilized cells	Low recovery and damage with stronger pipetting were reported for an earlier streptolysin O workflow; perfringolysin O assays cited here do not recover an isolated mitochondrial fraction
Osmotic swelling/osmotic assistance (Clayton and Shadel, 2014; Aksu‐Menges et al., 2025)	Low as a stand-alone method; useful as an adjunct	Moderate	Pre-treatment to reduce the mechanical force needed for Dounce or Potter-Elvehjem disruption	Excessive swelling can damage membranes; usually must be paired with mechanical disruption
Detergent-mediated membrane solubilization (Gurtubay et al., 1980; Nicholson and McMurray, 1986; Zhou et al., 2023)	High	High with standardized conditions	Mitochondrial protein extraction, membrane solubilization, immunoblotting, and sub-mitochondrial localization; not recommended for isolation of intact functional mitochondria	Broad membrane solubilization disrupts mitochondrial inner and outer membranes, resulting in loss of structural integrity and bioenergetic function
Microfluidic disruption (Rahman et al., 2018; Lin et al., 2019; Hsiao et al., 2018)	Low to moderate currently	High	Small-volume, controlled, standardized disruption of cells	Device complexity, limited sample throughput, and limited validation across tissue types
Automated mechanical dissociation (Gross et al., 2011; Franko et al., 2013; Preble et al., 2014)	High	High	Mammalian biopsies or workflows requiring rapid standardized tissue disruption	Device-specific conditions still need validation for each tissue and downstream application

*Processing efficiency refers to the approximate time, labor, and handling burden required to disrupt a sample before downstream mitochondrial enrichment or purification. It does not refer directly to final mitochondrial yield or purity. High efficiency indicates rapid disruption or compatibility with larger/multiple samples. Moderate efficiency indicates practical use but with greater dependence on sample type, manual handling, or optimization. Low efficiency indicates limited stand-alone usefulness, low throughput, or dependence on additional disruption or enrichment steps.

### 3.1 Physical disruption

Physical disruption methods release mitochondria through the application of mechanical forces that rupture the plasma membrane while minimizing damage to intracellular organelles. Depending on the technique employed, cell disruption may be achieved through mechanisms including shear stress, compressive forces, pressure differentials, cavitation, or abrasive forces (Goldberg, 2008). The magnitude and mode of these forces influence not only the efficiency of cell lysis but also the structural and functional integrity of the recovered mitochondria (Picard et al., 2011). Although additional cell disruption strategies have been described, the methods discussed below represent the most widely adopted physical approaches for mitochondrial isolation across both discovery and translational research. The following sections outline the principles underlying each technique and then discuss their applications in mitochondrial isolation.

#### 3.1.1 Dounce homogenization

Dounce homogenization is a widely used physical disruption method for isolating mitochondria from mammalian cells and soft tissues (Dounce et al., 1955; Acín-Pérez et al., 2023; Clayton and Shadel, 2014). Developed by Alexander L. Dounce for the isolation of intact intracellular organelles, the method was designed to mechanically disrupt cells while minimizing damage to subcellular structures (Dounce et al., 1955). The homogenizer consists of a glass tube fitted with two glass pestles of different clearances, commonly referred to as the loose (A) and tight (B) pestles. During homogenization, repeated strokes generate compressive and shear forces that rupture the plasma membrane and release intracellular organelles while helping preserve mitochondrial integrity (Acín-Pérez et al., 2023; Clayton and Shadel, 2014).

The extent of homogenization is an important determinant of mitochondrial recovery, and protocols are typically optimized according to the tissue or cell type being processed (Acín-Pérez et al., 2023; Clayton and Shadel, 2014). Modern mitochondrial isolation protocols commonly use the loose pestle for initial disruption followed by the tight pestle to achieve more complete cell lysis, although the number of strokes varies depending on the biological source (Acín-Pérez et al., 2023). For example, Acín-Pérez et al. recommend six strokes with both the loose and tight pestles for mouse brain, whereas heart tissue requires approximately twenty strokes with each pestle because of its denser structure (Acín-Pérez et al., 2023). Consequently, Dounce homogenization is most commonly applied to cultured mammalian cells and relatively soft tissues, where controlled mechanical disruption is required before mitochondrial purification by differential centrifugation (Acín-Pérez et al., 2023; Clayton and Shadel, 2014).

#### 3.1.2 Potter-Elvehjem homogenization

Potter–Elvehjem homogenization is a commonly used physical disruption method for isolating mitochondria from tissue samples, particularly when a more controlled motor-driven homogenization step is preferred (Liao et al., 2020; Frezza et al., 2007; Clayton and Shadel, 2014). The method uses a glass tube and a rotating Teflon pestle to mechanically disrupt tissue through shear and compressive forces generated between the pestle and the homogenizer wall (Liao et al., 2020; Frezza et al., 2007; Clayton and Shadel, 2014).Unlike fully manual glass-on-glass homogenization, the Potter–Elvehjem system can apply a more consistent rotational force, making it useful for processing tissues that require repeated and controlled homogenization before mitochondrial enrichment (Liao et al., 2020; Frezza et al., 2007).

In mitochondrial isolation workflows, Potter–Elvehjem homogenization is typically performed in a cold isotonic isolation buffer before differential centrifugation (Liao et al., 2020; Frezza et al., 2007; Clayton and Shadel, 2014). The number of passes, rotation speed, and tissue-to-buffer ratio are important variables because the homogenization step must disrupt cells efficiently without excessive mechanical damage to mitochondria (Liao et al., 2020; Clayton and Shadel, 2014). For example, Liao et al. describe the use of a Potter–Elvehjem homogenizer for tissue disruption and noted that multiple passes are required to achieve maximal cell disruption (Liao et al., 2020). Similarly, Frezza et al. used glass/Teflon Potter–Elvehjem homogenizers for mitochondrial isolation from mouse liver and skeletal muscle, with different homogenizer sizes selected according to whether the starting material was cells or tissue (Frezza et al., 2007).

Potter–Elvehjem homogenization is especially relevant for tissues such as liver, skeletal muscle, heart, kidney, and other solid organs where tissue architecture requires more sustained mechanical disruption than cultured cells alone (Liao et al., 2020; Frezza et al., 2007; Clayton and Shadel, 2014; Gross et al., 2011). However, as with other physical disruption methods, the intensity of homogenization must be adjusted to the biological source. Excessive or prolonged homogenization can compromise mitochondrial integrity, whereas insufficient homogenization can reduce mitochondrial recovery by leaving cells or tissue fragments incompletely disrupted (Liao et al., 2020; Clayton and Shadel, 2014; Gross et al., 2011). Therefore, Potter–Elvehjem homogenization is best understood as a controlled tissue disruption method that is widely incorporated into mitochondrial isolation workflows, particularly when paired with differential centrifugation or density-based purification.

#### 3.1.3 Rotor-stator homogenization

Rotor–Stator homogenization is an instrument-based physical disruption method that uses a rapidly rotating probe within a stationary outer element to generate strong shear forces for tissue disruption (Goldberg, 2008). In mitochondrial isolation workflows, Rotor–Stator systems are most relevant for tissues that are difficult to disrupt using manual glass pestle-based homogenization alone. Although many mitochondrial isolation studies rely on Dounce or Potter–Elvehjem homogenization, instrument-based disruption has been used to improve the consistency of tissue breakdown, particularly when processing mechanically resistant samples (Preble et al., 2014).

Rotor–Stator systems apply stronger mechanical forces than Dounce or Potter–Elvehjem homogenization, so disruption conditions must be carefully controlled to limit mitochondrial damage (Goldberg, 2008). Protocols using Rotor–Stator instruments typically apply brief homogenization times and low-speed settings, reflecting the need to disrupt tissue efficiently while minimizing mechanical and thermal stress during sample processing (QIAGEN, 2011). Instrument-based homogenization may also improve consistency when processing tissue samples, as standardized mechanical dissociation can reduce some of the variability associated with manual homogenization (Preble et al., 2014).

The available mitochondrial literature more commonly describes Rotor–Stator homogenization in protocols and commercial isolation workflows than in studies directly evaluating the method. As a result, it should be discussed as a useful but less well-characterized disruption strategy for mitochondrial isolation. Its main relevance is as an initial tissue disruption approach for fibrous or mechanically resistant samples, while later purification steps such as differential centrifugation, filtration, or density-gradient centrifugation remain necessary to enrich the mitochondrial fraction (Preble et al., 2014; QIAGEN, 2011).

#### 3.1.4 French press/French pressure cell

French press disruption, also referred to as French pressure cell disruption, is a pressure-based physical method that disrupts cells by forcing a suspension through a narrow valve under high pressure (French and Milner, 1955; Jaschke et al., 2009). As the sample exits the press, the rapid pressure drop and shear forces generated at the valve promote rupture of the plasma membrane or cell wall (French and Milner, 1955; Jaschke et al., 2009). Compared with pestle-based homogenization, the French press is most often associated with microorganisms, yeast, algae, and other mechanically resistant samples, although it has also been used in earlier mitochondrial isolation protocols from platelets and yeast (Watson et al., 1970; Fukami and Salganicoff, 1973).

In mitochondrial isolation, the French press is most relevant when the starting material is difficult to disrupt by gentle manual homogenization alone. For example, Watson et al. used low-pressure French press disruption to isolate mitochondrial structures from anaerobically grown *Saccharomyces cerevisiae*, reporting that approximately 90% of cells could be broken by passage through the press at 500 psi in sorbitol-EDTA buffer (Watson et al., 1970). The method has also been applied to platelet mitochondrial isolation, where nagarse-treated platelets were disrupted using a modified French pressure cell at approximately 800 psi (Fukami and Salganicoff, 1973). More recently, French press disruption has been used in organelle isolation from the diatom *Thalassiosira pseudonana*, where controlled pressure was important for cell rupture and preservation of organelle structure (Schober et al., 2019).

Although the French press can be useful for mechanically resistant samples, pressure settings require careful optimization. Excessive pressure can damage organelles, whereas insufficient pressure may leave cells incompletely disrupted. Schober et al. found that a constant working pressure of 90 MPa was important for effective cell rupture and organelle preservation in *T. pseudonana*, while also noting that other rupture approaches, including osmotic shock, enzymatic digestion, and glass bead homogenization, were unsuccessful for that system (Schober et al., 2019). Therefore, the French press should be considered a specialized physical disruption method for samples requiring pressure-based lysis rather than a general first-line approach for all mitochondrial isolation workflows.

#### 3.1.5 Nitrogen cavitation

Nitrogen cavitation is a pressure-based physical disruption method in which cells or tissues are equilibrated with nitrogen gas under high pressure and then rapidly decompressed to rupture cellular membranes. The method was introduced for pressure homogenization of mammalian tissues by Hunter and Commerford, who described nitrogen decompression to disrupt cells while preserving subcellular structures (Hunter and Commerford, 1961). Unlike pestle-based homogenization, nitrogen cavitation does not rely on direct grinding or repeated manual strokes. Instead, nitrogen dissolves into the sample under pressure and forms expanding gas bubbles during decompression, generating mechanical forces that disrupt the plasma membrane and release intracellular organelles (Hunter and Commerford, 1961; Simpson, 2010).

In mitochondrial isolation, nitrogen cavitation is often used when gentle and relatively uniform disruption is desired. Kristian et al. applied nitrogen cavitation to primary neuronal and astrocyte cultures and reported that the method produced mitochondria with high respiratory control, supporting its use for functional mitochondrial studies in sensitive cell types (Kristián et al., 2006). More recently, Younis et al. used nitrogen cavitation to isolate mitochondria from murine skeletal muscle and C2C12 cells, describing the recovery of intact, respiring mitochondria without the need for purification gradients or ultracentrifugation (Younis et al., 2023). These studies suggest that nitrogen cavitation can be useful for mitochondrial isolation when preservation of respiratory function is a priority, particularly in samples where excessive shear or grinding may compromise mitochondrial integrity.

The effectiveness of nitrogen cavitation depends strongly on pressure, equilibration time, decompression conditions, and the biological source being processed (Hunter and Commerford, 1961; Kristián et al., 2006; Simpson, 2010; Younis et al., 2023). If the applied pressure is too low, cell disruption may be incomplete; if it is too high, organelles may become fragile or damaged during decompression (Hunter and Commerford, 1961; Simpson, 2010). For this reason, nitrogen cavitation protocols typically require empirical optimization for each sample type. Although the method can provide controlled and reproducible disruption, it also requires specialized pressurized equipment and may be less practical for small laboratories or high-throughput workflows compared with simpler homogenization-based approaches.

#### 3.1.6 Bead beating/bead milling

Bead beating, or bead milling when performed in a mill-based format, is a physical disruption method that uses repeated collisions between cells and small beads to mechanically rupture resistant samples (Mogren et al., 1974; Schütte et al., 1983; Woodrow and Quirk, 1982). Early bead-mill studies focused on the mechanical disintegration of microorganisms, where rotating discs or agitators moved glass beads through a cell suspension to break yeast and bacterial cells (Mogren et al., 1974; Schütte et al., 1983). Unlike Dounce or Potter–Elvehjem homogenization, which are most often applied to mammalian cells and soft tissues, bead-based disruption is particularly relevant for organisms with rigid cell walls, including yeast, fungi, bacteria, and microalgae (Mogren et al., 1974; Schütte et al., 1983; Woodrow and Quirk, 1982; Postma et al., 2017). In these samples, abrasive and impact forces generated by glass, ceramic, or metal beads can disrupt the cell wall and plasma membrane, allowing intracellular material, including mitochondria, to be released for downstream fractionation (Schütte et al., 1983; Woodrow and Quirk, 1982; Postma et al., 2017; Diekert et al., 2001).

In mitochondrial isolation, bead-based disruption has mainly been used for yeast and other resistant cell types that are difficult to lyse by gentle homogenization alone (Diekert et al., 2001; Mendoza-Martínez et al., 2026). Diekert et al. describe glass bead lysis as a rapid method for preparing crude mitochondrial fractions from *Saccharomyces cerevisiae*, while also distinguishing this approach from gentler spheroplast-based methods used to obtain more refined mitochondrial preparations (Diekert et al., 2001). More recent protocols have adapted glass bead disruption to preserve mitochondrial-associated proteins, such as Atg32, during yeast mitochondrial isolation by performing lysis under cold, protease-inhibited conditions (Mendoza-Martínez et al., 2026). These examples suggest that bead-based disruption can be useful when cell wall disruption is the main barrier to mitochondrial recovery, especially in yeast models where rapid mechanical lysis may be preferred over enzymatic spheroplasting for specific downstream analyses (Diekert et al., 2001; Mendoza-Martínez et al., 2026).

The same mechanical forces that make bead beating effective can also make it difficult to use to achieve highly intact mitochondrial preparations. Bead size, bead loading, agitation speed, processing time, sample concentration, and temperature all influence the extent of disruption and the degree of damage to the recovered material (Mogren et al., 1974; Schütte et al., 1983; Woodrow and Quirk, 1982; Postma et al., 2017; Mendoza-Martínez et al., 2026). For this reason, bead-based mitochondrial protocols generally use short disruption cycles, cold buffers, and protease inhibitors to reduce sample heating and preserve mitochondrial proteins during lysis (Mendoza-Martínez et al., 2026). Overall, bead beating should be discussed as a specialized physical disruption strategy for resistant cell types rather than a general first-line approach for mammalian mitochondrial isolation.

#### 3.1.7 Sonication

Sonication is a physical disruption method that uses high-frequency sound waves to break open cells through acoustic cavitation. The biological effects of high-frequency sound waves were first described by Wood and Loomis in 1927, who showed that intense sound waves could produce physical and biological disruption in exposed samples (Wood and Loomis, 1927). In modern cell disruption workflows, sonication uses an ultrasonic probe or bath to generate rapid pressure changes in liquid, leading to the formation and collapse of cavitation bubbles. This produces localized shear forces, pressure changes, and heat that can rupture cellular membranes and release intracellular contents (Rahman et al., 2018; Shehadul Islam et al., 2017).

In mitochondrial isolation, sonication has been used more selectively than Dounce, Potter–Elvehjem, or nitrogen cavitation because the method can be harsh for membrane-bound organelles. Chaiyarit et al. compared multiple disruption methods for *Candida albicans* before mitochondrial isolation and found that sonication was effective for mitochondrial protein extraction and proteomic analysis (Chaiyarit and Thongboonkerd, 2009). However, this type of application differs from protocols designed to recover highly intact, functional mitochondria for respiration assays or transplantation-related studies. Since sonication can fragment membranes, generate heat, and disrupt organelle structure, it is generally more suitable for applications where mitochondrial protein recovery is the primary goal rather than preservation of intact mitochondrial function (Rahman et al., 2018; Shehadul Islam et al., 2017; Chaiyarit and Thongboonkerd, 2009).

The main variables that influence sonication include power, frequency, pulse duration, total processing time, sample volume, temperature control, and probe position (Rahman et al., 2018; Shehadul Islam et al., 2017). Excessive sonication can damage the biological material being isolated, while insufficient sonication may leave cells incompletely disrupted. For this reason, sonication-based protocols typically use short pulses, cooling intervals, and samples maintained on ice to reduce heating and uncontrolled mechanical damage (Rahman et al., 2018; Shehadul Islam et al., 2017). Overall, sonication should be discussed as a powerful but comparatively harsh disruption method that may be useful for resistant samples or protein-focused mitochondrial analyses, but it is generally less appropriate when the goal is to isolate structurally intact and bioenergetically functional mitochondria.

#### 3.1.8 Electroporation

Electroporation is a physical disruption method that uses short, high-voltage electrical pulses to increase the permeability of the plasma membrane. The applied electric field induces a transmembrane potential that promotes the formation of aqueous pores within the lipid bilayer. Under moderate conditions, these pores may reseal after treatment, whereas stronger or prolonged electrical pulses can cause irreversible membrane disruption and cell lysis (Tsong, 1991; Kotnik et al., 2019).

Electroporation has received limited investigation as a method for mitochondrial isolation. Bahnemann et al. compared electroporation with Dounce homogenization, digitonin permeabilization, and sonication for disrupting Chinese hamster ovary cells before mitochondrial recovery by differential centrifugation. Although electroporation permitted mitochondrial recovery, sonication produced the highest mitochondrial yield under the conditions tested (Bahnemann et al., 2014). The outcome of electroporation is influenced by factors including electric-field strength, pulse duration, pulse number or frequency, waveform, electrode configuration, and the properties of the cells being processed (Kotnik et al., 2019). These parameters would require careful optimization to disrupt the plasma membrane without compromising mitochondrial membranes or bioenergetic function. Therefore, electroporation represents a potentially controllable and reproducible physical disruption method, but its suitability for isolating intact, functional mitochondria remains insufficiently characterized.

### 3.2 Chemical and enzymatic strategies for mitochondrial release

Chemical and enzymatic strategies can contribute to mitochondrial isolation by preparing cells or tissues for subsequent disruption or by acting directly on cellular membranes (Gross et al., 2011; Okutani et al., 2025). Pretreatment approaches, including proteolytic digestion, hypotonic swelling, and saponin treatment, facilitate mitochondrial release by weakening tissue architecture or increasing plasma membrane susceptibility to mechanical disruption (Gross et al., 2011; Clayton and Shadel, 2014; Okutani et al., 2025). In these workflows, pretreatment is followed by mechanical disruption rather than used as a stand-alone isolation method (Gross et al., 2011; Clayton and Shadel, 2014; Okutani et al., 2025). Detergent-based approaches can also facilitate mitochondrial release by solubilizing the plasma membrane, although their effects can extend to mitochondrial membranes (Okutani et al., 2025; Gurtubay et al., 1980).

The effectiveness of these approaches depends on reagent concentration, exposure conditions, and the biological source (Gross et al., 2011; Okutani et al., 2025). Insufficient membrane weakening may limit mitochondrial recovery, whereas excessive exposure to membrane-active reagents can compromise mitochondrial membrane integrity (Okutani et al., 2025). Proteolytic treatment can also affect the recovery and detection of specific mitochondrial proteins (Koncsos et al., 2018). Chemical and enzymatic conditions should therefore be selected and optimized according to both the starting material and the intended downstream application (Gross et al., 2011; Okutani et al., 2025; Koncsos et al., 2018).

#### 3.2.1 Enzymatic pretreatment for mitochondrial release

Enzymatic pretreatment facilitates mitochondrial release by digesting structural components that hinder tissue dissociation or cell disruption. The enzymes used depend on the biological source. Proteases assist the digestion of tissue-associated proteins in animal samples, whereas cell-wall-degrading enzymes prepare yeast cells for subsequent disruption (Gross et al., 2011; Lai et al., 2019; Gregg et al., 2009). These treatments are generally combined with mechanical disruption and subsequent mitochondrial separation.

Serine proteases are commonly used in mitochondrial isolation from heart and skeletal muscle, where digestion of myofibrillar proteins helps release mitochondria retained within the contractile apparatus (Palmer et al., 1977; Koncsos et al., 2018). These include subtilisins, with Nagarse as a commonly used example, and trypsin (Koncsos et al., 2018; Acín-Pérez et al., 2023). Nagarse has been used to pretreat minced skeletal muscle before mechanical disruption, while trypsin-based protocols have been described for skeletal muscle and can also be applied to heart tissue (Gross et al., 2011; Garcia-Cazarin et al., 2011).

Metalloproteases, including collagenases and neutral proteases such as dispase, assist tissue dissociation by digesting extracellular matrix components and tissue attachments. Collagenase IV has been used to dissociate mouse liver tissue before cell disruption and mitochondrial sorting, while dispase has been incorporated into skeletal muscle isolation workflows to facilitate tissue dissociation before homogenization (MacDonald et al., 2019; Lai et al., 2019).

Cell-wall-degrading enzymes, including β-glucanases, are particularly relevant to mitochondrial isolation from yeast. These enzymes digest glucan components of the cell wall, producing spheroplasts that are more susceptible to mechanical disruption. For example, Zymolyase, an enzyme preparation with β-1,3-glucanase activity, has been used to generate yeast spheroplasts before homogenization and mitochondrial purification (Gregg et al., 2009).

Enzymatic treatment can improve access to mitochondria that are difficult to release mechanically and, in some workflows, reduce non-mitochondrial protein contamination (Palmer et al., 1977; Kras et al., 2016). Protease treatment, however, can alter the recovery and detection of mitochondrial proteins even when respiratory function is preserved, potentially affecting downstream protein analyses (Koncsos et al., 2018). Enzyme selection, concentration, and exposure conditions therefore require optimization for the starting material, with appropriate termination of treatment and assessment of mitochondrial properties relevant to the intended downstream application (Acín-Pérez et al., 2023; Koncsos et al., 2018).

#### 3.2.2 Saponin-assisted mitochondrial release

Saponins are membrane-active glycosides that interact with membrane lipids and can alter membrane organization and permeability. Their effects vary with the saponin preparation and membrane composition, with cholesterol contributing to their membrane interactions (Korchowiec et al., 2015; Sudji et al., 2015). In mitochondrial isolation, saponin treatment has been used to weaken the plasma membrane and facilitate mitochondrial release during subsequent mechanical disruption (Okutani et al., 2025).

Digitonin, a steroidal saponin, has been used for this purpose in cultured cells and in enzymatically pretreated skeletal muscle and liver samples. Treatment was followed by removal of excess reagent and gentle pipetting to release mitochondria for collection by differential centrifugation (Okutani et al., 2025). This approach combined chemical membrane weakening with gentle mechanical handling and yielded mitochondria with preserved ATP-generating function and greater outer membrane integrity than the homogenization-based preparation (Okutani et al., 2025).

A limitation of saponin treatment is that membrane selectivity depends on the treatment conditions. Increasing digitonin concentrations can permeabilize mitochondrial membranes as well as the plasma membrane (Niklas et al., 2011). In the digitonin-assisted isolation method, brief exposure at low temperature and removal of excess reagent before cell disruption were used to limit mitochondrial exposure (Okutani et al., 2025). These findings highlight the importance of controlling treatment conditions when using saponins to support mitochondrial release.

#### 3.2.3 Cholesterol-dependent cytolysins

Cholesterol-dependent cytolysins are pore-forming proteins that can permeabilize the plasma membrane, with applications in mitochondrial isolation and permeabilized-cell assays. For mitochondrial isolation, Shibata et al. used streptolysin O pretreatment followed by gentle pipetting to disrupt cells and release mitochondria. The elongated mitochondria recovered using this approach retained intact inner and outer membranes and ATP-generating activity, although some spherical mitochondria showed outer-membrane damage (Shibata et al., 2015). Streptolysin O pretreatment followed by pipetting and differential centrifugation has also been used to isolate mitochondria from several mammalian cell types, including mesenchymal stromal cells, hepatocellular carcinoma cells, myoblasts, and induced pluripotent stem cells (Endo et al., 2026). However, Okutani et al. reported low yields with their earlier streptolysin O approach and mitochondrial damage when more vigorous pipetting was used to increase recovery, highlighting the balance between mitochondrial release and membrane preservation (Okutani et al., 2025). Another member of this class, perfringolysin O, has been used to selectively permeabilize cells for mitochondrial respiration measurements. In these protocols, mitochondria remain within permeabilized cells rather than being recovered as an isolated fraction (Salabei et al., 2014).

#### 3.2.4 Osmotic pretreatment for mitochondrial release

>Osmotic disruption is a chemical or buffer-based approach that uses osmotic conditions to promote cell swelling or disrupt subcellular membranes. In mitochondrial isolation workflows, hypotonic buffers can be used to swell cells before mechanical rupture, making the plasma membrane easier to disrupt with methods such as Dounce or Potter–Elvehjem homogenization (Clayton and Shadel, 2014). In these protocols, osmotic swelling is combined with subsequent mechanical disruption and differential centrifugation to release and recover mitochondria from cultured cells (Clayton and Shadel, 2014).

Osmotic swelling serves as a pretreatment before cell breakage. For example, Clayton and Shadel describe a mitochondrial isolation protocol in which cultured cells are first swollen in hypotonic buffer and then ruptured using a tight-fitting Dounce or Potter–Elvehjem pestle before mitochondrial recovery by differential centrifugation (Clayton and Shadel, 2014). More recent work has also incorporated hypotonic swelling into mitochondrial isolation from primary human skeletal myoblasts, where it was combined with Dounce homogenization, differential centrifugation, and subsequent purification steps to reduce cellular contamination in the isolated mitochondrial fraction. This method was evaluated for proteomic studies, without testing mitochondrial respiratory function (Aksu‐Menges et al., 2025).

The use of hypotonic swelling before cell disruption should be distinguished from its application directly to isolated mitochondria. In the latter context, osmotic shock can be used to generate mitoplasts through disruption of the outer mitochondrial membrane, rather than preserve intact mitochondria (Ma et al., 2023). In the cell isolation workflows described above, osmotic swelling therefore functions as a preparatory step within a broader isolation procedure (Clayton and Shadel, 2014; Aksu‐Menges et al., 2025).

#### 3.2.5 Detergent-mediated membrane solubilization

Detergents such as Triton X-100, NP-40, Tween-20, and SDS have been used to solubilize mitochondrial membranes or extract mitochondrial proteins, with their effects depending on the reagent and treatment conditions (Gurtubay et al., 1980; Alam, 2003; Pou De Crescenzo et al., 2001; Li et al., 2002). These applications include the extraction of mitochondrial components after isolation. For example, NP-40 was used to solubilize isolated human placental mitochondria for analysis of mitochondrial DNA–protein complexes, whereas Tween-20 combined with NaCl solubilized an inner-membrane-associated NAD+ kinase from oat mitochondria (Alam, 2003; Pou De Crescenzo et al., 2001). SDS has also been used following digitonin fractionation to extract mitochondrial proteins for immunoblotting (Li et al., 2002).

Triton X-100 provides a useful example of how detergent solubilization can affect mitochondrial membranes. Gurtubay et al. showed that Triton X-100 solubilizes components of both rat liver mitochondrial inner and outer membranes, with effects on membrane organization and enzyme activities depending on the treatment conditions (Gurtubay et al., 1980). Similarly, Zhou et al. use Triton X-100 after mitochondrial isolation to permit proteinase K access to otherwise protected mitochondrial proteins for sub-mitochondrial localization assays (Zhou et al., 2023). These examples illustrate the value of detergents for mitochondrial component analysis, while showing why conditions that solubilize mitochondrial membranes are incompatible with recovering structurally intact mitochondria.

### 3.3 Emerging cell disruption technologies

Emerging cell disruption technologies aim to improve the control, reproducibility, and scalability of mitochondrial release while reducing the variability associated with manual homogenization (Rahman et al., 2018; Lin et al., 2019). Although conventional methods such as Dounce, Potter–Elvehjem, detergent permeabilization, and nitrogen cavitation remain widely used, newer approaches are being developed to standardize membrane disruption, reduce operator dependence, and preserve mitochondrial integrity during release from cells or tissues (Rahman et al., 2018; Lin et al., 2019). These methods remain less established than traditional workflows, but they may be useful for applications that require small sample volumes, rapid processing, or more controlled disruption conditions.

#### 3.3.1 Microfluidic disruption platforms

Microfluidic disruption platforms use microscale channels, constrictions, or junction geometries to apply controlled mechanical stress to cells (Rahman et al., 2018; Lin et al., 2019; Hsiao et al., 2018). Instead of disrupting samples through repeated manual strokes or bulk homogenization, these systems expose cells to defined hydrodynamic forces that can be tuned to rupture the plasma membrane while aiming to preserve mitochondrial membranes (Rahman et al., 2018; Lin et al., 2019). This approach is attractive for mitochondrial isolation because it may reduce operator variability and allow membrane damage to be controlled more precisely than in conventional mechanical homogenization (Rahman et al., 2018; Lin et al., 2019; Hsiao et al., 2018).

Rahman et al. developed a microscale cell shredder that used hydrodynamic stress in a cross-junction geometry to selectively disrupt cellular membranes while preserving mitochondrial integrity (Rahman et al., 2018). In this system, cells were first swollen in a hypotonic buffer and then passed through a microfluidic device under optimized flow conditions (Rahman et al., 2018). The authors reported that the microscale cell shredder produced more functional mitochondria than Dounce homogenization or a commercial mitochondria isolation kit under the tested conditions, supporting the potential of microfluidic disruption as an alternative to traditional homogenization-based methods (Rahman et al., 2018).

Other microfluidic devices have also been developed to extract mitochondria under chemical-free or additive-free conditions (Lin et al., 2019; Hsiao et al., 2018). Lin et al. described a centrifugal microfluidic device designed to extract healthy mitochondria using a standard laboratory centrifuge, with the device incorporating a microfluidic component for efficient cell membrane disruption (Lin et al., 2019). Similarly, Hsiao et al. proposed a chip-based microfluidic device to extract functional mitochondria without chemical detergents, which may be useful when downstream applications require minimal reagent exposure (Hsiao et al., 2018). However, these platforms remain relatively early-stage and have generally been tested in limited sample types or small-scale formats (Rahman et al., 2018; Hsiao et al., 2018). Further validation is needed to determine whether microfluidic disruption can be broadly applied to diverse tissues and larger-scale mitochondrial isolation workflows.

#### 3.3.2 Automated mechanical dissociation systems

Automated mechanical dissociation systems are emerging as alternatives to manual homogenization because they use programmed disruption cycles or device-controlled tissue processing to standardize the release of mitochondria from cells or tissues (Preble et al., 2014; Franko et al., 2013; Gross et al., 2011). In mitochondrial isolation workflows, these systems are mainly being explored to reduce operator-dependent variability, improve processing consistency, and support rapid tissue disruption before downstream mitochondrial separation (Preble et al., 2014; Franko et al., 2013; Gross et al., 2011; Liao et al., 2020).

Preble et al. described a rapid mitochondrial isolation workflow for transplantation applications in which manual homogenization was replaced with a commercial tissue dissociator (Preble et al., 2014). In this approach, mammalian tissue biopsies were processed using a standardized homogenization cycle before mitochondrial recovery, reducing the need for manual tissue grinding and allowing rapid sample preparation (Preble et al., 2014). The authors noted that this standardized cycle allows more uniform and consistent tissue homogenization than manual homogenization, which is important for workflows where isolation time and reproducibility are major concerns (Preble et al., 2014).

Semi-automated disruption has also been applied to mechanically resistant tissues. Gross et al. described methods for isolating functional mitochondria from rat kidney and skeletal muscle without manual homogenization, using a Pressure Cycling Technology (PCT) Shredder and pressure-based processing to disrupt tissue (Gross et al., 2011). The PCT Shredder was described as a mechanical grinder that rapidly breaks up tissue without significant risk of over-homogenization, while pressure cycling was used to support mitochondrial release from the disrupted tissue (Gross et al., 2011). Although automated mechanical dissociation systems may improve consistency, they still require optimization for each tissue type because excessive processing can damage mitochondria, while incomplete disruption can reduce mitochondrial release (Gross et al., 2011; Liao et al., 2020).

## 4 Mitochondrial separation and purification methods

Following cell disruption, mitochondria must be separated from unbroken cells, nuclei, plasma membrane fragments, cytosolic proteins, lysosomes, peroxisomes, endoplasmic reticulum, and other subcellular contaminants. Separation and purification methods are therefore central determinants of mitochondrial yield, purity, structural integrity, and functional quality (Frezza et al., 2007; Liao et al., 2020). Most mitochondrial isolation workflows rely on differences in size, density, sedimentation behavior, or surface protein expression to enrich mitochondria from crude cell or tissue homogenates (Frezza et al., 2007; Liao et al., 2020; Franko et al., 2013). Although differential centrifugation remains the most widely used approach, additional purification steps such as density-gradient centrifugation, filtration, or immunoaffinity-based enrichment may be incorporated when higher purity, greater specificity, or improved standardization is required (Frezza et al., 2007; Liao et al., 2020; Franko et al., 2013).

### 4.1 Differential centrifugation

Differential centrifugation is the most widely used method for enriching mitochondria from crude cell or tissue homogenates (Frezza et al., 2007; Liao et al., 2020; Clayton and Shadel, 2014). After cell disruption, the homogenate is subjected to sequential centrifugation steps that separate cellular components based on differences in size, mass, and sedimentation behavior (Frezza et al., 2007; Liao et al., 2020; Clayton and Shadel, 2014; Graham, 1999a). Low-speed centrifugation is first used to remove unbroken cells, nuclei, and large debris, while subsequent higher-speed centrifugation pellets mitochondria from the post-nuclear supernatant (Frezza et al., 2007; Liao et al., 2020; Clayton and Shadel, 2014; Graham, 1999a). The resulting mitochondrial pellet can then be washed and resuspended for downstream structural, biochemical, or functional analyses (Frezza et al., 2007; Liao et al., 2020; Clayton and Shadel, 2014).

A major advantage of differential centrifugation is its simplicity and broad applicability. The method requires standard laboratory centrifugation equipment and can be adapted to cultured cells, soft tissues, and solid organs by modifying the homogenization conditions, centrifugation speeds, and wash steps (Frezza et al., 2007; Liao et al., 2020; Clayton and Shadel, 2014; Graham, 1999a). Frezza et al. describe differential centrifugation-based workflows for isolating functional mitochondria from mouse liver, skeletal muscle, and cultured fibroblasts, while Liao et al. similarly note that most mitochondrial isolation methods rely on differential centrifugation as the core separation strategy (Frezza et al., 2007; Liao et al., 2020). Differential centrifugation has therefore become the conventional starting point for mitochondrial isolation across many experimental systems, including cultured cells, animal tissues, and specialized subcellular fractionation workflows (Frezza et al., 2007; Liao et al., 2020; Clayton and Shadel, 2014; Graham, 1999a; Wieckowski et al., 2009).

Despite its widespread use, differential centrifugation often produces mitochondrial fractions that are enriched rather than fully purified (Liao et al., 2020; Graham, 1999a; Wieckowski et al., 2009; Franko et al., 2013). Because mitochondria can overlap in size and sedimentation behavior with lysosomes, peroxisomes, endoplasmic reticulum fragments, plasma membrane vesicles, and other subcellular components, contamination can remain in the final pellet (Liao et al., 2020; Graham, 1999a; Wieckowski et al., 2009; Franko et al., 2013). This is particularly important when downstream analyses require high purity, because contaminating organelles or membrane fragments can influence measurements of mitochondrial proteins, metabolism, or function (Graham, 1999a; Wieckowski et al., 2009; Franko et al., 2013). Repeated washing steps can reduce some contaminants, but additional purification methods, such as density-gradient centrifugation or immunoaffinity-based enrichment, may be required when higher purity is necessary (Liao et al., 2020; Wieckowski et al., 2009; Franko et al., 2013). Therefore, differential centrifugation is best viewed as a foundational mitochondrial enrichment method that balances accessibility and recovery, but may require additional purification depending on the downstream application.

### 4.2 Density-gradient centrifugation

Density-gradient centrifugation is a purification method used to separate mitochondria from other subcellular components based on differences in buoyant density. Unlike differential centrifugation, which enriches mitochondria primarily by sequential pelleting, density-gradient centrifugation places the crude mitochondrial fraction onto a gradient medium, such as sucrose, Percoll^®^, Nycodenz™, or iodixanol, allowing mitochondria to band separately from contaminants during centrifugation (Graham, 1999b; Kristian, 2010; Liao et al., 2020). This approach is commonly used when a higher-purity mitochondrial preparation is needed after initial enrichment by differential centrifugation (Graham, 1999b; Kristian, 2010; Liao et al., 2020; Anunciado-Koza et al., 2023).

The main advantage of density-gradient centrifugation is improved separation of mitochondria from organelles and membrane fragments that may co-pellet during differential centrifugation. Crude mitochondrial fractions are often contaminated by lysosomes, peroxisomes, Golgi membranes, endoplasmic reticulum fragments, and other vesicular material, making further purification important for applications that require clean mitochondrial fractions (Graham, 1999b; Liao et al., 2020; Anunciado-Koza et al., 2023). Percoll^®^ gradients are commonly used for mitochondrial purification from tissues such as brain and skeletal muscle, where they can help separate mitochondria from myelin, synaptosomes, debris, or other tissue-specific contaminants (Kristian, 2010; Anunciado-Koza et al., 2023). Sucrose and iodixanol-based gradients may also be used depending on the sample type and downstream application (Graham, 1999b; Liao et al., 2020).

Despite its advantages, density-gradient centrifugation adds time, handling steps, and technical complexity to the isolation workflow. Additional centrifugation and washing steps may reduce contamination, but they can also decrease mitochondrial recovery or expose mitochondria to prolonged processing conditions (Graham, 1999b; Kristian, 2010; Liao et al., 2020; Anunciado-Koza et al., 2023). Gradient media may also need to be removed carefully before downstream assays, especially when mitochondrial respiration, membrane potential, or biochemical measurements are planned (Liao et al., 2020). Therefore, density-gradient centrifugation is best viewed as a higher-purity purification step that is especially useful when contamination must be minimized, but it may not be necessary for every mitochondrial isolation workflow.

### 4.3 Filtration-based separation

Filtration-based separation uses porous membranes or mesh filters to remove large debris, intact cells, nuclei, and tissue fragments from crude homogenates while allowing smaller organelles, including mitochondria, to pass through or be retained depending on the filter design. In mitochondrial isolation workflows, filtration is often used after tissue disruption as a clarification or enrichment step rather than as a complete purification method on its own (Preble et al., 2014; Bodenstein et al., 2023). By reducing large contaminants before centrifugation or downstream enrichment, filtration can simplify the isolation workflow and may shorten processing time compared with repeated centrifugation steps (Preble et al., 2014; Bodenstein et al., 2023; McCully et al., 2009).

Filtration-based workflows have been especially emphasized in mitochondrial transplantation studies, where rapid preparation of fresh mitochondria is important. Preble et al. described a rapid mitochondrial isolation method in which tissue dissociation was followed by sequential filtration to remove large debris and enrich the mitochondrial fraction (Preble et al., 2014). In this workflow, filtration was used as an alternative to longer centrifugation-based purification steps, allowing mitochondria to be isolated from mammalian biopsies within a short processing window (Preble et al., 2014). Subsequent studies have further optimized differential filtration conditions for mitochondrial transplantation applications, including evaluation of filter pore size and sample preparation variables to improve mitochondrial recovery and viability (Bodenstein et al., 2023).

The main advantage of filtration-based separation is speed and simplicity. Because filtration can remove large debris without multiple lengthy centrifugation steps, it may be useful when mitochondria must be isolated rapidly for functional assays or transplantation-oriented workflows (Preble et al., 2014; Bodenstein et al., 2023; McCully et al., 2009). However, filtration alone is unlikely to provide the same level of purity as density-gradient centrifugation or immunoaffinity-based enrichment because smaller contaminants, vesicles, and organelle fragments may pass through the filter along with mitochondria (Preble et al., 2014; Bodenstein et al., 2023; McCully et al., 2009; Graham, 1999b). Therefore, filtration-based separation should be considered as a rapid enrichment or clarification strategy that can support mitochondrial isolation, but its suitability depends on whether the downstream application prioritizes speed, yield, purity, or functional preservation.

### 4.4 Immunoaffinity-based isolation

Immunoaffinity-based isolation uses antibodies against mitochondrial surface proteins to selectively capture mitochondria from crude cell or tissue homogenates. Instead of separating mitochondria only by size, density, or sedimentation behavior, this approach relies on recognition of proteins located on the outer mitochondrial membrane, such as TOM22 or TOM20 (Franko et al., 2013; Hornig-Do et al., 2009; Chen et al., 2016). In many workflows, antibodies are coupled to magnetic beads or microfluidic surfaces, allowing labelled mitochondria to be separated from other subcellular material using magnetic or affinity-based capture (Franko et al., 2013; Hornig-Do et al., 2009; Chen et al., 2016; Kayo et al., 2013).

This approach is useful when high specificity or purity is required. Franko et al. described the isolation of pure and functional mitochondria from mouse tissues using automated tissue disruption followed by anti-TOM22 magnetic bead enrichment (Franko et al., 2013). TOM22 is an appropriate target because it is part of the translocase of the outer mitochondrial membrane complex and is exposed on the mitochondrial surface, allowing intact mitochondria to be captured using antibody-conjugated beads (Franko et al., 2013; Hornig-Do et al., 2009). Beyond tissue-scale purification, immunoaffinity-based strategies have also been adapted for rapid analytical workflows, including mitochondrial immunocapture for metabolomic analysis and microfluidic antibody-based separation from small cell culture samples (Chen et al., 2016; Kayo et al., 2013).

The main advantage of immunoaffinity-based isolation is improved specificity compared with differential centrifugation alone. Because crude mitochondrial pellets can contain lysosomes, peroxisomes, endoplasmic reticulum fragments, and other vesicular contaminants, antibody-based enrichment can help produce cleaner mitochondrial fractions for downstream applications where purity is especially important (Franko et al., 2013; Chen et al., 2016; Kayo et al., 2013). However, immunoaffinity methods also have limitations. They can be more expensive, require access to suitable antibodies or magnetic bead systems, and may be less practical for large-scale mitochondrial recovery. Antibody binding and bead handling may also influence mitochondrial recovery or downstream compatibility depending on the application. Therefore, immunoaffinity-based isolation is best viewed as a high-specificity purification strategy, particularly useful for analytical workflows, rather than a universal replacement for conventional centrifugation-based methods.

## 5 Reporting framework for mitochondrial isolation

The success of a mitochondrial isolation method cannot be determined solely by the recovery of a visible pellet or the detection of mitochondrial markers. To support consistent evaluation and comparison of isolation methods, we propose a reporting framework organized around four complementary criteria: respiration, integrity, purity, and viability window. Reports should describe the amount of mitochondrial material recovered relative to the starting sample, the conditions used to assess each criterion, and the interval between isolation and assessment or use. These measurements should be interpreted together to establish whether a preparation is suitable for its intended downstream application. Reproducibility across independent isolations and preservation of mitochondrial quality during scale-up provide additional criteria for evaluating practical suitability. A broader comparison of the scalability and suitability of each disruption approach for research and potential clinical applications is provided in **Table 2**.

**Table 2:** Mitochondrial isolation methods: clinical scalability and translational potential.

Disruption approach	Scalability*	Research suitability	Translational/clinical potential**
Dounce homogenization (Dounce et al., 1955; Clayton and Shadel, 2014; Preble et al., 2014; Acín-Pérez et al., 2023)	Low. Best suited to small analytical samples. Increasing sample mass makes disruption more operator-dependent and harder to control without under- or over-homogenization.	Strong for small-scale research, especially cultured mammalian cells and relatively soft tissues.	Limited by manual handling, open processing, and difficulty validating operator-independent consistency for good manufacturing practices.
Potter-Elvehjem homogenization (Frezza et al., 2007; Clayton and Shadel, 2014)	Low to moderate. More controllable than manual Dounce homogenization, but scale-up remains limited by homogenizer volume, tissue density, pass number, and the risk of uneven tissue disruption.	Strong for research and process development; more standardizable than fully manual Dounce.	Requires enclosed or sterile-compatible processing, validated tissue-specific operating parameters, and consistent preservation of mitochondrial integrity and function.
Rotor-Stator homogenization (Idell-Wenger et al., 1982; Goldberg, 2008; Preble et al., 2014)	Moderate Adaptable to larger or fibrous tissue samples, but speed, processing time, sample heating, and shear intensity must be tightly controlled to preserve mitochondrial integrity.	Useful for scale-up and process development when rapid tissue breakdown is needed.	Requires strict control of shear, heat generation, containment, and post-disruption mitochondrial quality, particularly when processing fibrous or heterogeneous tissues.
French press/pressure cell (Watson et al., 1970; Fukami and Salganicoff, 1973)	Low to moderate. Scalable for suspended or mechanically resistant samples, but pressure, valve geometry, and passage number must be optimized to avoid incomplete disruption or organelle damage.	Specialized research method for yeast, algae, microorganisms, platelets, or other mechanically resistant sources.	Requires sterile pressure-compatible processing, validated pressure conditions, and evidence that mitochondrial integrity and function are preserved after pressure-based disruption.
Nitrogen cavitation (Kristián et al., 2006; Younis et al., 2023)	Moderate. Scalable within pressure-vessel capacity, but pressure, equilibration time, and decompression conditions must be optimized to preserve mitochondrial function.	Strong for functional research where preservation of respiration is important.	Requires specialized pressure equipment, sterile sample loading and recovery, equipment qualification, operator safety controls, and source-specific validation showing preserved mitochondrial integrity and bioenergetic function.
Bead beating/bead milling (Diekert et al., 2001; Mendoza-Martínez et al., 2026)	Moderate to high for resistant cells; low to moderate for intact mitochondria. The method can process larger or multiple samples, but bead size, agitation intensity, cycle duration, and heat generation can compromise mitochondrial quality.	Useful for resistant research models such as yeast, fungi, bacteria, and algae.	Limited by harsh impact forces, heat generation, debris burden, and the difficulty of validating intact functional mitochondrial recovery from mechanically disrupted material.
Sonication (Chaiyarit and Thongboonkerd, 2009; Shehadul Islam et al., 2017)	Low to moderate. Rapidly scalable for cell disruption or protein extraction, but acoustic cavitation, heat generation, and uneven energy distribution limit its suitability for intact functional mitochondrial recovery.	Appropriate for resistant samples or mitochondrial protein/proteomics-focused research.	Limited by acoustic cavitation, heat generation, membrane fragmentation, and the difficulty of demonstrating consistent preservation of intact mitochondrial function.
Electroporation (Tsong, 1991; Kotnik et al., 2019; Bahnemann et al., 2014)	Moderate. Scalable in principle through programmable pulse parameters, but field strength, pulse duration, pulse number, and electrode configuration would require validation for intact mitochondrial recovery.	Potentially useful for research optimization because electric-field strength, pulse duration, and pulse number are adjustable.	Requires substantial validation showing that electrical permeabilization releases mitochondria without compromising membrane potential, mitochondrial integrity, or downstream functional criteria.
Enzymatic pretreatment/assisted disruption (Gross et al., 2011; Garcia-Cazarin et al., 2011; Gregg et al., 2009; Koncsos et al., 2018; Lai et al., 2019; MacDonald et al., 2019; Preble et al., 2014)	Moderate. Enzyme quantities and buffer volumes can be adjusted to sample size, but digestion and subsequent mechanical disruption must remain controlled. Larger tissue inputs can complicate processing and reduce mitochondrial recovery.	Useful for muscle preparations, tissue dissociation, and yeast cell-wall digestion. Enzyme selection and treatment conditions depend on the source and downstream analyses.	Incorporated into rapid transplantation-oriented workflows. Further development should address enzyme activity, removal or inactivation, and consistent preservation of mitochondrial function and proteins.
Saponin-assisted mitochondrial release (Salabei et al., 2014; Niklas et al., 2011; Okutani et al., 2025)	Moderate. Scalable if reagent concentration, exposure time, temperature, and mixing are controlled, but membrane selectivity can be lost if conditions are not optimized.	Strong for mechanistic and functional research involving selective plasma-membrane permeabilization.	Requires strict reagent qualification, control of concentration and exposure time, residual reagent testing, removal validation, membrane-selectivity validation, and compatibility with downstream good manufacturing practices.
Cholesterol-dependent cytolysin pretreatment (Shibata et al., 2015; Salabei et al., 2014; Okutani et al., 2025; Endo et al., 2026)	Low. Published isolation workflows depend on controlled pretreatment and pipetting, with limited validation at larger inputs. Increasing pipetting intensity to improve recovery can compromise mitochondrial integrity.	Useful for mitochondrial isolation from cultured mammalian cells using streptolysin O. Perfringolysin O supports respiration measurements in permeabilized cells rather than recovery of isolated mitochondria.	Further validation is needed to establish consistent recovery and membrane preservation. Potential processing considerations include reagent removal, residual cytolysin activity, and control of subsequent mechanical disruption.
Osmotic swelling/osmotic assistance (Clayton and Shadel, 2014; Aksu‐Menges et al., 2025)	Low as a stand-alone approach; moderate as an adjunct. Useful for reducing the mechanical force required during disruption, but buffer conditions and swelling time must be controlled to avoid membrane damage.	Supportive research step to reduce mechanical force before Dounce or Potter-Elvehjem disruption.	Depends on validation of buffer composition, osmotic exposure time, sterility, and compatibility with the full downstream disruption and mitochondrial isolation workflow.
Detergent-mediated membrane solubilization (Gurtubay et al., 1980; Alam, 2003; Pou De Crescenzo et al., 2001; Li et al., 2002; Zhou et al., 2023)	Not applicable for intact mitochondrial recovery. Detergent extraction can accommodate larger samples, but membrane solubilization limits its suitability for producing intact mitochondria regardless of processing capacity.	Suitable for mitochondrial component extraction, membrane-associated enzyme analysis, immunoblotting, and sub-mitochondrial localization.	Membrane-solubilizing conditions are unsuitable for preparing intact functional mitochondria for transplantation. These reagents remain useful for analytical testing after isolation.
Microfluidic disruption (Rahman et al., 2018; Lin et al., 2019; Hsiao et al., 2018)	Low currently; potentially high with engineering. Highly controllable at small volumes, but scale-up is limited by device throughput, clogging risk, and validation across larger or heterogeneous tissue inputs.	Promising for controlled small-volume research and method development.	Device-contained processing could support standardization, but broader validation would be required for larger inputs, heterogeneous tissues, clogging risk, sterility, device materials, and preservation of mitochondrial function.
Automated mechanical dissociation (Gross et al., 2011; Franko et al., 2013; Preble et al., 2014)	Moderate to high Well suited for repeated or larger-scale tissue disruption because device-controlled programs can standardize processing, although tissue-specific settings still require validation.	Strong for research and translational process development, especially when reproducibility is important.	More feasible than manual homogenization because device-controlled processing can support standardization; however, sterile consumables, device qualification, validated programs, source-material ethics, and mitochondrial quality controls would still be required.

*Scalability refers to how easily a method can be expanded from small experimental samples to larger sample volumes or higher throughput without substantially reducing yield, purity, reproducibility, or product quality.

**Translational/clinical potential represents an assessment based on published method characteristics and anticipated requirements for clinical-scale processing; most disruption approaches have not been validated for clinical manufacturing.

### 5.1 Respiration

Respiratory assessment should establish whether isolated mitochondria retain the capacity to support substrate oxidation and couple respiration to ATP production. This can be evaluated using complementary measurements of oxygen consumption, respiratory coupling, and ATP-generating capacity, selected according to the biological source and intended downstream application. Relevant measurements include responses to ADP, selected respiratory-chain or ATP synthase inhibitors, and uncouplers. These approaches provide information about different aspects of mitochondrial function, with the choice of reagents guided by the respiratory pathway or activity being assessed (Lanza and Nair, 2009; Rogers et al., 2011).

Reporting should identify the respiratory conditions examined and include the measured rates alongside any derived indices. For example, when the respiratory control ratio (RCR) is used, its calculation should specify the respiratory states being compared (Lanza and Nair, 2009; Rogers et al., 2011). Responses to inhibitors should likewise be interpreted according to their targets: respiratory-chain inhibitors can help establish the contribution of particular electron transport pathways, whereas ATP synthase inhibitors can help assess coupling to phosphorylation (Rogers et al., 2011). Where ATP-generating capacity is central to the intended application, direct measurement of ATP production can provide complementary evidence. Measuring ATP production over time distinguishes the capacity to generate ATP from the amount already present in the preparation (Drew and Leeuwenburgh, 2003).

Regardless of the approach selected, reports should describe the assay conditions and normalization method sufficiently to support interpretation and comparison. Relevant details include the substrates and reagents supplied, their concentrations, assay temperature, buffer composition, and amount of mitochondrial material analyzed. These conditions influence measured respiratory activity, and mitochondrial loading requires particular attention in microplate assays (Lanza and Nair, 2009; Rogers et al., 2011). The emphasis should therefore be on demonstrating respiratory function under clearly defined conditions, using measurements appropriate to the intended use of the preparation.

### 5.2 Integrity

Integrity assessment should establish whether isolated mitochondria retain their structural organization and membrane properties. Relevant approaches include microscopy, assessment of compartment-specific protein retention, membrane-permeability assays, and membrane-potential measurements. These approaches provide complementary information because isolation can alter mitochondrial morphology and function, while mitochondrial markers may remain detectable in damaged preparations (Picard et al., 2011; Okutani et al., 2025). Reports should identify the features examined and provide evidence appropriate to the intended downstream application.

Structural assessment can include transmission electron microscopy (TEM) to examine outer and inner membrane continuity, cristae organization, matrix appearance, and swelling. Confocal fluorescence imaging can complement TEM by assessing mitochondrial morphology and the distribution of fluorescent labels, allowing comparison of organelle shape, size, and fragmentation across preparations (Picard et al., 2011). Reports should specify which structural features were evaluated and the imaging method used. Imaging can also be complemented by analysis of compartment-specific proteins, such as TOM20 or VDAC for the outer membrane, COX IV for the inner membrane, cytochrome c or adenylate kinase 2 for the intermembrane space, and citrate synthase for the matrix. Their retention or loss can support assessment of compartment preservation, although their presence alone does not demonstrate intact membranes (Okutani et al., 2025).

Membrane integrity can also be assessed through biochemical approaches. Options include measuring cytochrome c retention or release, or examining whether added cytochrome c stimulates respiration, which can indicate outer-membrane damage accompanied by loss of endogenous cytochrome c (Lanza and Nair, 2009). Comparing citrate synthase activity before and after deliberate membrane disruption provides another measure of membrane integrity, with increased substrate accessibility following disruption allowing assessment of the previously enclosed enzyme (Boutagy et al., 2015). Protease protection assays offer complementary information by comparing the accessibility of selected mitochondrial proteins to digestion before and after detergent treatment (Zhou et al., 2023). Reporting should specify the compartment assessed, the intact and disrupted controls used, and the measured response rather than describing the preparation simply as “intact.”

Membrane-potential measurements provide information about polarization across the inner mitochondrial membrane. Available probes include tetramethylrhodamine methyl ester (TMRM), tetramethylrhodamine ethyl ester (TMRE), rhodamine 123, and JC-1. TMRM, TMRE, and rhodamine 123 accumulate in polarized mitochondria, while JC-1 exhibits potential-dependent accumulation and formation of red-fluorescent aggregates, allowing assessment of aggregate and monomer fluorescence (Scaduto and Grotyohann, 1999; Reers et al., 1991). These signals require probe-specific interpretation. In TMRM non-quench mode, greater polarization generally increases mitochondrial fluorescence. At higher, self-quenching concentrations, depolarization can instead produce a transient fluorescence increase as the dye redistributes and becomes unquenched. The observed response depends on the concentration regime and the location and timing of fluorescence measurement (Scaduto and Grotyohann, 1999; Ward et al., 2000). Reports should therefore describe dye concentration, loading conditions, measurement mode, and acquisition settings, together with the response to a depolarizing control such as FCCP.

Preserved membrane potential alone does not establish ATP-generating capacity. When respiratory-chain activity is impaired and ATP is available, complex V can operate in reverse, hydrolyzing ATP to sustain polarization. A preparation retaining a strong potential-dependent signal may therefore be consuming ATP rather than producing it (Ward et al., 2000; Hagen et al., 2025). Membrane-potential measurements should consequently be interpreted alongside evidence of respiratory coupling or ATP production, as described in Section 5.1. Where ATP synthase direction is uncertain, its response to inhibition provides one additional option: depolarization following oligomycin is consistent with prior ATP hydrolysis supporting membrane potential (Ward et al., 2000). The reporting emphasis should be on complementary evidence of structural preservation, membrane integrity, and bioenergetic function, rather than treating any single marker or fluorescence signal as proof of suitability for therapeutic use.

### 5.3 Purity

Purity assessment should establish the extent of mitochondrial enrichment and the presence of non-mitochondrial material in the recovered fraction. Detection of mitochondrial markers alone does not establish purity; it should be accompanied by assessment of contaminants relevant to the starting material and isolation method. Approaches can include immunoblotting, microscopy, and proteomic analysis, selected according to the intended downstream application (Franko et al., 2013; Liao et al., 2020). Reports should describe both the evidence for enrichment and the contaminants examined.

Immunoblotting provides one approach for comparing mitochondrial and non-mitochondrial markers across the starting material, intermediate fractions, and final preparation. Marker selection should reflect the biological source. For example, TOM20, histone H3, and β-tubulin have been used to evaluate mitochondrial enrichment and nuclear and cytoplasmic contamination in preparations from primary human skeletal myoblasts (Aksu‐Menges et al., 2025). In yeast preparations, mitochondrial markers such as Tom70, porin, and Kgd1 can be assessed alongside Sec61, hexokinase, and Nyv1 to examine contamination from the endoplasmic reticulum, cytosol, and vacuole, respectively (Liao et al., 2020). Reports should identify the markers used, the fractions compared, and the loading and quantification methods so that enrichment and contaminant depletion can be interpreted.

Complementary analyses can provide information beyond a selected marker panel. TEM can reveal co-isolated organelles and membrane material, while proteomic analysis can assess the representation of mitochondrial and non-mitochondrial proteins across the preparation (Franko et al., 2013). Reports should specify how purity was assessed and, where quantified, how it was calculated.

Interpretation should also account for material associated with mitochondria before isolation. Mitochondria-associated endoplasmic reticulum membranes can accompany mitochondrial fractions and require additional separation when the objective is to examine mitochondria independently of these contacts (Wieckowski et al., 2009). Reports should clarify whether such associated material was retained or depleted and how this relates to the intended application. Purity should ultimately be considered alongside recovery, integrity, and respiration, with conclusions limited to the contaminants and properties actually assessed.

### 5.4 Viability window

The viability window refers to the interval after isolation during which a mitochondrial preparation retains the properties required for its intended application. Within this reporting framework, the emphasis is on the time required to complete isolation and quality assessment, and whether the measured quality is maintained until the preparation is used. Rapid isolation methods have been developed to recover respiration-competent mitochondria while shortening processing time, highlighting the practical importance of reporting both isolation duration and functional outcomes (Preble et al., 2014).

Reports should state the interval between sample collection, completion of isolation, quality assessment, and downstream use. Relevant assessments can include respiration, ATP production, membrane potential, and membrane integrity, selected according to the intended application (Lanza and Nair, 2009; Rogers et al., 2011). When a preparation is used after a delay, measurements at relevant time points can help establish whether its required properties remain preserved. Any intervening handling conditions should be described so that the interval between assessment and use can be interpreted.

The reported viability window should be based on the properties and time points actually examined. Detection of mitochondrial markers or retained membrane potential alone does not establish continued ATP-generating capacity, because polarization can persist through ATP consumption (Ward et al., 2000). The aim is therefore to establish that mitochondria remain suitable at the point of use, with any decline in the measured structural or functional properties reported alongside the isolation timeline.

### 5.5 Reproducibility and scalability

Reproducibility refers to whether an isolation method consistently recovers mitochondria with comparable yield, purity, integrity, and function across independent preparations. Assessment can draw on the complementary measurements described above, with results reported across separate isolations rather than only repeated measurements of the same preparation. Studies combining mitochondrial recovery, imaging, biochemical analysis, and respiration provide examples of how multiple properties can be evaluated together (Franko et al., 2013; Preble et al., 2014). Reports should identify the number of independent preparations, describe variability in the measured outcomes, and indicate whether consistency was examined across different operators or experimental runs.

Scalability concerns whether the method can accommodate larger amounts of starting material or increased sample throughput while maintaining mitochondrial quality. These capabilities should be distinguished: processing several samples in parallel does not necessarily demonstrate that an individual isolation can accommodate a larger tissue mass or cell number. For example, Franko et al. developed an automated tissue-disruption and magnetic-enrichment approach intended to support reproducible processing of parallel samples (Franko et al., 2013). Within this framework, reports should specify the range of starting quantities and number of samples processed, together with any changes to disruption conditions, reagent volumes, purification steps, or processing time.

Where scalability is evaluated, mitochondrial recovery should be considered alongside respiration, integrity, purity, and the interval between isolation and use. Increased recovery alone should not be taken as evidence that preparation quality has been maintained. Practical considerations can include equipment requirements, manual handling, processing time, and compatibility with the intended workflow. These features are illustrated by approaches using automated disruption or rapid tissue dissociation and filtration, although their suitability at a different scale requires evaluation under the proposed conditions (Franko et al., 2013; Preble et al., 2014). Claims of reproducibility and scalability should therefore reflect the conditions actually tested and the consistency of the resulting mitochondrial preparations.

## 6 Current challenges and opportunities for improvement

A major challenge in mitochondrial isolation is the lack of standardization across protocols. Although most workflows involve cell disruption, mitochondrial enrichment, and washing or purification, the specific disruption method, buffer composition, centrifugation speed, processing time, temperature, and purification strategy vary widely between studies examining different cell and tissue sources (Liao et al., 2020; Picard et al., 2011; Frezza et al., 2007). This variability makes it difficult to determine whether differences in mitochondrial yield, purity, morphology, or respiration reflect the biological source being processed or the isolation procedure itself (Liao et al., 2020; Picard et al., 2011). Greater standardization in reporting starting material, disruption conditions, centrifugation parameters, buffer composition, temperature, yield, purity, membrane integrity, and functional readouts is needed to improve reproducibility and allow more meaningful comparison between isolation methods (Liao et al., 2020; Picard et al., 2011; Lanza and Nair, 2009).

Another persistent challenge is balancing mitochondrial recovery with organelle quality. More aggressive disruption methods can improve mitochondrial release but may also increase membrane damage, alter mitochondrial morphology, or impair respiration (Picard et al., 2011; Lanza and Nair, 2009). In contrast, gentler approaches may better preserve mitochondrial structure and function but can result in incomplete cell disruption and lower recovery (Liao et al., 2020; Picard et al., 2011). This trade-off is important because mitochondrial isolation cannot be judged by yield alone. A preparation with high protein content or a visible mitochondrial pellet may still contain damaged mitochondria, fragmented membranes, or non-mitochondrial contaminants (Picard et al., 2011; Lanza and Nair, 2009; Graham, 1999b). Therefore, isolation methods should be evaluated using complementary measures of yield, purity, structural integrity, membrane potential, oxygen consumption, ATP production, and respiratory control rather than relying on a single endpoint (Lanza and Nair, 2009; Rogers et al., 2011; Pesta and Gnaiger, 2012).

Purity also remains a major limitation of many conventional workflows. Differential centrifugation is widely used because it is simple and broadly applicable, but crude mitochondrial fractions can contain lysosomes, peroxisomes, endoplasmic reticulum fragments, plasma membrane vesicles, and other subcellular contaminants (Liao et al., 2020; Graham, 1999b). These contaminants can influence downstream measurements of mitochondrial proteins, metabolites, enzyme activity, or respiration, particularly when the goal is biochemical or functional analysis (Lanza and Nair, 2009; Graham, 1999b; Rogers et al., 2011). In a clinical setting, impurities may affect both the safety and therapeutic potency of the mitochondrial preparation. Co-isolated nuclear material, cellular debris, and other organelles may introduce immunogenic or biologically active components, while damaged mitochondria and mitochondrial fragments can release mitochondrial DNA and other damage-associated molecular patterns that promote sterile inflammation and immune activation (Bodenstein et al., 2024).

Density-gradient centrifugation and immunoaffinity-based enrichment can improve mitochondrial purity, but they also add time, technical complexity, cost, and potential loss of mitochondrial recovery (Graham, 1999b; Franko et al., 2013). Future improvements should therefore focus on selecting purification strategies based on the downstream application, rather than assuming that the highest-purity method is always the most appropriate.

Source-specific optimization is another important opportunity for improvement. Different biological sources vary in mitochondrial abundance, tissue architecture, membrane composition, fragility, and resistance to disruption, meaning that a protocol optimized for one sample type may not work well for another (Liao et al., 2020; Frezza et al., 2007). Cultured mammalian cells and soft tissues may be suitable for gentler homogenization approaches, whereas fibrous tissues, yeast, microorganisms, or mechanically resistant samples may require stronger disruption methods (Liao et al., 2020; Frezza et al., 2007; Diekert et al., 2001). Similarly, blood-derived sources such as platelets may require different optimization strategies than solid tissues because of differences in mitochondrial content, sample volume, activation risk, and processing requirements. Future protocols should therefore be optimized and reported according to the specific starting material rather than relying on universal isolation conditions.

For translational applications, additional challenges include scalability, processing time, sterility, operator dependence, and compatibility with preservation or cryopreservation. Rapid workflows using standardized tissue dissociation and differential filtration have been developed to reduce isolation time and improve consistency for transplantation-oriented applications (Preble et al., 2014). Emerging approaches such as microfluidic disruption platforms and digitonin-assisted intact mitochondrial isolation also show potential for improving control over mitochondrial release while limiting mechanical or chemical damage (Rahman et al., 2018; Okutani et al., 2025). However, many of these approaches remain limited by small-scale validation, device-specific conditions, or uncertainty about performance across diverse tissues and larger starting volumes (Preble et al., 2014; Rahman et al., 2018; Okutani et al., 2025). For mitochondrial transplantation or other therapeutic applications, future isolation methods will need to preserve mitochondrial structure and bioenergetic function while also being compatible with closed or semi-closed processing systems, sterility requirements, and preservation workflows (Preble et al., 2014; Okutani et al., 2025).

These processing constraints become particularly important when mitochondrial preparation must be coordinated with urgent reperfusion. An analysis of thrombectomy trials found that each additional hour to reperfusion was associated with a 5.2-percentage-point reduction in functional independence among patients who achieved substantial reperfusion (Saver et al., 2016). Although this finding concerns reperfusion rather than mitochondrial transplantation, it explains why mitochondrial preparation should not delay restoration of blood flow. The initial pediatric cardiac report described mitochondrial harvest and isolation taking 20–30 minutes from a 6 × 6-mm rectus abdominis biopsy (Emani et al., 2017). In the Phase 1 stroke study, isolation from approximately 0.1 g of skeletal muscle included separate 10-minute digestion and centrifugation steps, alongside homogenization, filtration, and handling. Time constraints also restricted quality assessment, with samples preserved for subsequent TEM analysis rather than completing ultrastructural evaluation before administration (Walker et al., 2026). The recovered preparation was divided between administration and quality testing, illustrating the need to accommodate both requirements when starting material is limited. These clinical workflows highlight the competing demands of rapid processing, sufficient mitochondrial recovery, and adequate assessment before use. However, the reported preparation times are not established therapeutic cutoffs, and the optimal interval between ischemic injury and mitochondrial administration remains unresolved.

Overall, future progress in mitochondrial isolation will likely depend on combining optimized disruption methods with appropriate purification strategies and standardized quality-control assays. Improvements should prioritize not only higher yield, but also preservation of membrane integrity, respiratory function, purity, reproducibility, and scalability (Picard et al., 2011; Lanza and Nair, 2009; Rogers et al., 2011; Pesta and Gnaiger, 2012). Establishing source-specific benchmarks and reporting standards would help clarify which isolation methods are best suited for different applications, including biochemical analysis, functional studies, preservation research, and future therapeutic development.

## 7 Conclusion

Mitochondrial isolation remains a critical step in both mitochondrial research and the development of mitochondrial-based therapeutic strategies. Although many isolation methods have been established, no single approach is optimal for all sample types or downstream applications. Physical disruption, chemical permeabilization, emerging microfluidic approaches, differential centrifugation, density-gradient purification, filtration, and immunoaffinity-based enrichment each offer distinct advantages and limitations. Methods that increase mitochondrial release may also introduce mechanical, chemical, osmotic, or processing-related stress that compromises membrane integrity, morphology, or bioenergetic function (Picard et al., 2011; Liao et al., 2020). For this reason, mitochondrial isolation should not be evaluated by yield alone. Instead, successful isolation requires complementary assessment of mitochondrial purity, structural integrity, membrane potential, respiratory capacity, ATP production, and reproducibility (Lanza and Nair, 2009; Rogers et al., 2011). These quality-control measures are especially important when isolated mitochondria are intended for functional studies, preservation research, or transplantation-oriented applications.

Future progress will depend on improving standardization, source-specific optimization, and scalability. Clear reporting of starting material, disruption conditions, centrifugation parameters, purification steps, buffer composition, temperature, processing time, and quality-control readouts would make studies easier to compare and reproduce. Emerging approaches, including standardized tissue dissociation, microfluidic disruption, and digitonin-assisted intact mitochondrial isolation, provide opportunities to improve control over mitochondrial release while reducing operator variability and processing-related damage (Preble et al., 2014; Okutani et al., 2025). However, these methods still require broader validation across different tissues, sample volumes, and downstream applications.

Overall, robust mitochondrial isolation requires more than the recovery of a mitochondrial fraction. The goal should be to obtain mitochondria that are sufficiently abundant, pure, structurally intact, membrane-polarized, functionally active, and reproducible across preparations. Establishing standardized benchmarks for mitochondrial quality and developing scalable workflows compatible with preservation and clinical translation will be essential for advancing both experimental mitochondrial biology and future mitochondrial therapies.
